# Apical Malignant Pleural Mesothelioma Presenting as Horner’s Syndrome: A Case Report

**DOI:** 10.7759/cureus.94484

**Published:** 2025-10-13

**Authors:** Shruthi Mankal, Chris Jacobs

**Affiliations:** 1 Medicine, Great Western Hospital National Health Service (NHS) Foundation Trust, Swindon, GBR; 2 Faculty of Life Sciences and Medicine, King's College London, London, GBR; 3 Psychology, University of Bath, Bath, GBR

**Keywords:** apical lung tumor, asbestos exposure, brachial plexopathy, horner’s syndrome, malignant pleural mesothelioma (mpm), pancoast syndrome

## Abstract

Horner’s syndrome is a well-recognized presentation of apical lung tumors, though rarely associated with malignant pleural mesothelioma (MPM), with only a few cases documented. We report the case of an 82-year-old man with persistent right-sided chest pain who developed unilateral right-sided ptosis, miosis and a sunken appearance to the eye, in the context of known asbestos exposure.

Initial chest radiograph was reported as normal; however, subsequent computed tomography revealed an irregular right apical pleural mass measuring 7.0×4.3×7.5 cm. Biopsy with immunohistochemistry confirmed sarcomatoid MPM. This case report illustrates apical MPM mimicking Pancoast syndrome through compression of the upper thoracic sympathetic chain, resulting in preganglionic Horner's syndrome characterized by unilateral ptosis, miosis and anhidrosis ipsilateral to the lesion.

In patients with known asbestos exposure, persistent respiratory symptoms should prompt a thorough investigation for MPM. The presence of preganglionic Horner’s syndrome alone, or in combination with brachial plexopathy (arm pain, paresthesia or weakness, shoulder pain, or chest pain) - termed Pancoast syndrome, should heighten clinical suspicion of apical MPM. Cross-sectional imaging should be considered in all cases with a high index of suspicion, even if initial radiographs appear normal. Early recognition may facilitate timely diagnosis. This report contributes valuable insight to the limited literature documenting the association between apical MPM and pre-ganglionic Horner's syndrome.

## Introduction

Malignant pleural mesothelioma (MPM) is a rare, aggressive malignancy with the highest global incidence of 29 cases per million in the United Kingdom and Australia [1]. Approximately 80% of cases are associated with prior asbestos exposure, and the characteristic long latency period between exposure and clinical presentation continues to drive rising incidence [2]. Early clinical manifestations, including dyspnea, chest pain and cough, are non-specific and may contribute to delayed diagnosis, with a median survival of 10.3 months from diagnosis [2,3].

Horner’s syndrome is characterized by unilateral ptosis, miosis and sometimes, facial anhidrosis caused by damage to the ipsilateral oculosympathetic pathway. This syndrome can arise from various etiologies including intracranial hemorrhage, mass lesions or vascular pathology affecting the internal carotid artery [4]. Importantly, in the context of thoracic malignancies, the presence of Horner’s syndrome can alert clinicians to potential tumor involvement of the lung apex. While Horner’s syndrome is a well-recognized presentation of apical lung malignancies, it is rarely associated with MPM, with a limited number of cases documented [5-7]. We present a case of apical MPM presenting with Horner's syndrome. This report elucidates the anatomical basis for this uncommon presentation and discusses diagnostic considerations that may facilitate earlier recognition and diagnosis.

## Case presentation

**Figure 1:**
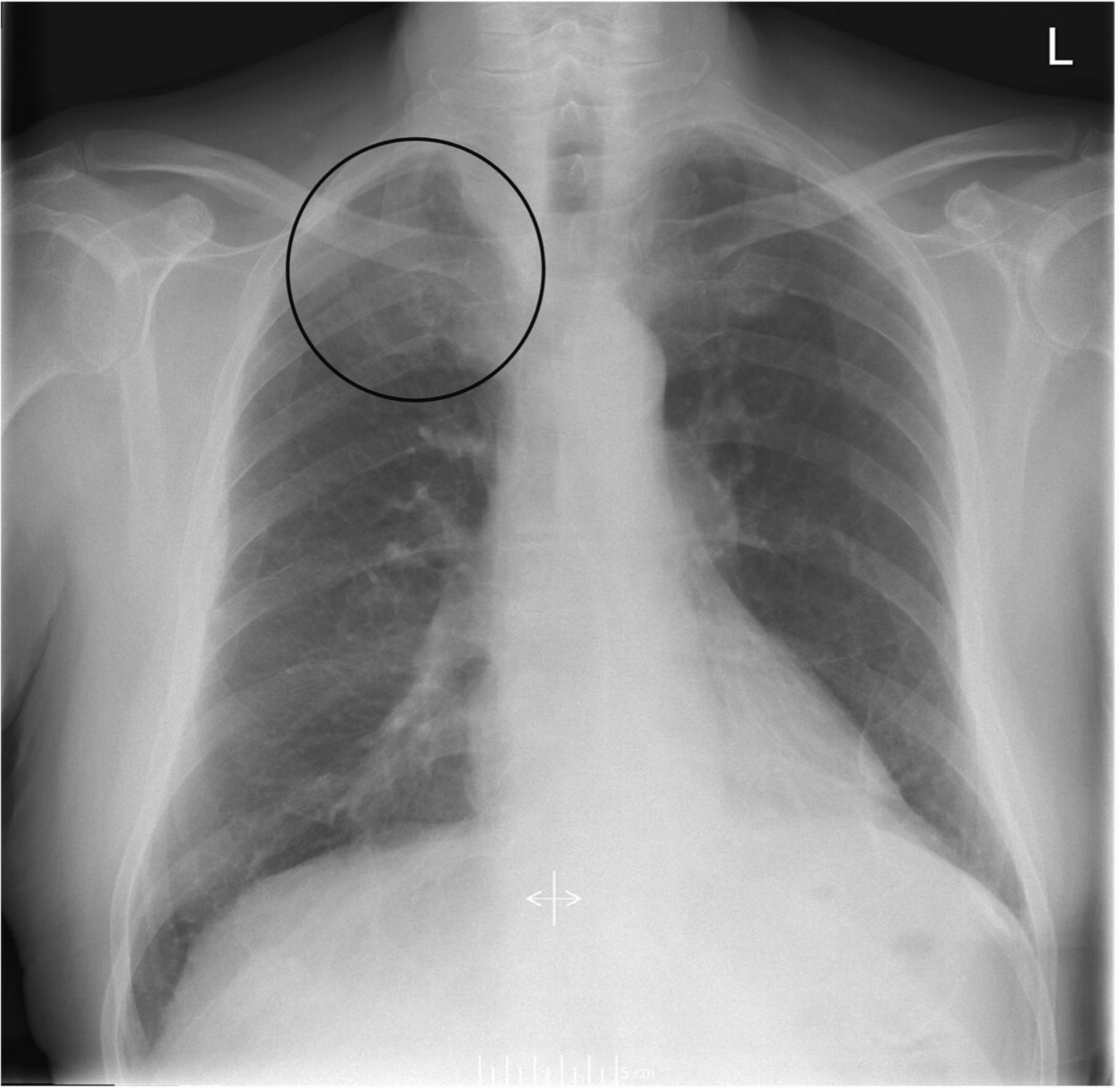
Chest radiograph with subtle opacification in the right upper zone (circle)

**Figure 2:**
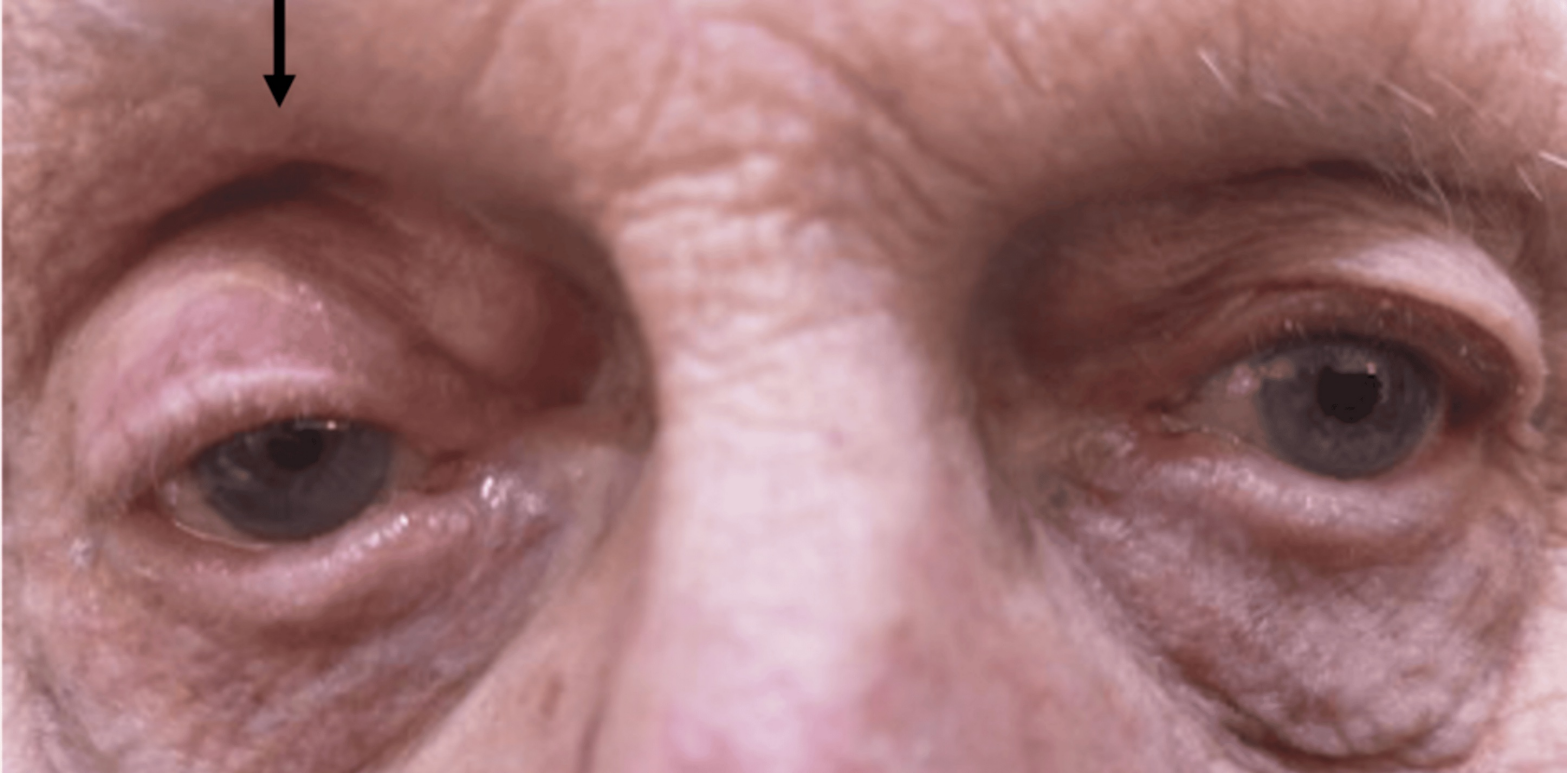
Patient presented with Horner’s syndrome as evidenced by right-sided (arrow) ptosis, miosis and a sunken appearance to the eye

An 82-year-old man presented to primary care with a two-month history of right-sided chest pain around the right nipple, having been initially treated for a presumed lower respiratory tract infection. Symptoms persisted despite empirical treatment with oral antibiotics, and a chest radiograph obtained during this period was reported as normal (Figure 1). Two months later, the patient developed a tremor affecting his right arm. Physical examination at this time revealed the characteristic features of Horner’s syndrome: unilateral ptosis, miosis and a sunken appearance of the right eye (Figure 2). During subsequent hospital review a few weeks later, the patient developed a dry cough, hoarse voice, and noted unintentional weight loss. His ongoing chest pain remained poorly controlled despite regular paracetamol and oral morphine.

The patient’s medical history included hypertension, a reversed hemicolectomy, and a total knee replacement. Of particular relevance was his 20-pack-year smoking history and occupational asbestos exposure during his employment as a railway fitter. Given these risk factors and the clinical findings, an urgent respiratory referral was made under the two-week wait pathway.

**Figure 3:**
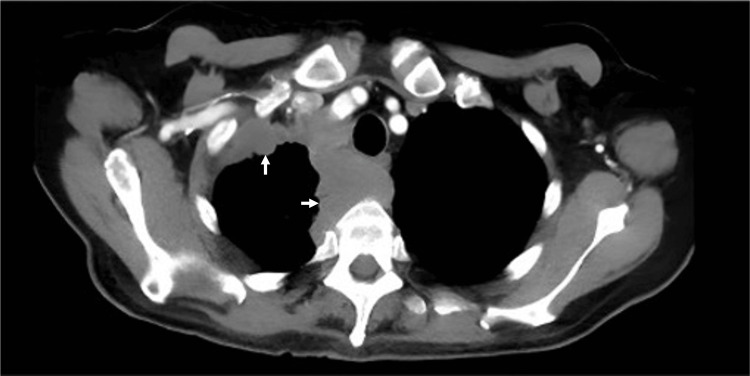
Computed tomography of the thorax (CT-thorax) demonstrating an irregular right apical pleural mass (white arrows) measuring 7.0 × 4.3 × 7.5 cm maximally, extending from the anterior to posterior mediastinum

Subsequent computed tomography of the thorax revealed an irregular, right apical pleural mass (Figure 3). The report noted a lobulated mass measuring 7x4.3x7.5 cm, with the anterior and posterior borders extending to the pleural surfaces, displacement of the superior aspect of the superior vena cava and associated pretracheal lymphadenopathy.

Bronchoscopy was performed one week later, followed by a biopsy and histopathology. Microscopy demonstrated cellular atypia, with the presence of spindle cells and sclerotic fibrous tissue. Immunohistochemical analysis revealed the presence of spindle cells positive for vimentin, MCK and WT1, alongside focal positivity for thrombomodulin and mesothelin, while remaining negative for calretinin. These findings are consistent with sarcomatoid mesothelioma, confirming the diagnosis of malignant pleural mesothelioma [8]. Following a discussion of the lung cancer multidisciplinary team (MDT), the patient was deemed unfit for chemotherapy. He was managed palliatively, dying nine months from symptom onset secondary to metastases.

## Discussion

Apical MPM may clinically mimic Pancoast tumors and present as a Pancoast syndrome, though the frequency of apical MPM presentation and associated Pancoast syndrome remains poorly documented. Beyond a single additional case reported by Chave et al. [9], recent literature specifically describing MPM with Pancoast syndrome is scarce, likely reflecting both the rarity of MPM and the infrequency of apical invasion, which tends to occur in more advanced disease. The anatomical basis for Horner's syndrome in apical thoracic malignancies involves compression of the upper thoracic sympathetic chain, which disrupts the second-order neurons of the oculosympathetic pathway as they traverse the apex of the lung [4,10,11]. This disruption results in a preganglionic Horner's syndrome, characterized by the classic triad of unilateral ptosis, miosis, and anhidrosis ipsilateral to the lesion [4]. Notably, anhidrosis was not documented in this case, though is frequently underreported by patients due to modern temperature-controlled spaces [12].

The postganglionic sympathetic fibers exit at the C8, T1, and T2 spinal nerve roots, hence disruption at this level may cause a brachial plexopathy with associated neurological manifestations including arm pain, paresthesia or weakness, shoulder pain, or chest pain as reported in this case [4,12]. The symptoms of preganglionic Horner’s syndrome coupled with manifestations of brachial plexopathy are collectively termed Pancoast syndrome [12]. The unilateral arm tremor observed in this case represents an atypical finding not classically associated with Pancoast syndrome. However, in the absence of other identifiable causes, this may represent a pseudo-tremor secondary to muscle atrophy [12]. This atrophy may result from nerve root involvement, although this mechanism has not been documented in the literature.

Whilst cases of MPM presenting with Pancoast syndrome have been reported in the literature, such presentations remain uncommon [5-7]. In this patient, the persistence of respiratory symptoms and chest pain, together with the development of preganglionic Horner’s syndrome and a history of asbestos exposure prompted further investigation by the general practitioner. The early manifestations of MPM are typically non-specific, with dyspnea, chest pain, cough and weight loss being the most frequent symptoms at initial diagnosis [3,12]. The persistence of these symptoms in patients with established risk factors, particularly known asbestos exposure, should prompt clinicians to maintain a low threshold for further investigation.

When evaluating preganglionic Horner's syndrome, it is important to consider the differential diagnosis of apical thoracic malignancies. Non-small cell lung cancer and adenoid cystic carcinoma are the most common malignancies associated with preganglionic Horner’s syndrome, hence should remain differentials [12]. The presence of preganglionic Horner's syndrome, with or without the brachial plexopathy characteristic of Pancoast syndrome, should prompt immediate and thorough investigation for underlying malignancy involving the lung apex, pleural surfaces, or brachial plexus. Such clinical presentations warrant urgent imaging to facilitate early diagnosis [12].

Although chest radiography was performed promptly, subtleties in findings may lead to apical lung tumors being underreported on plain films, as evidenced in the present case and other reports [13,14]. Not only does this underscore the importance of radiologist review of chest radiographs [14], but also suggests computed tomography (CT) of the thorax should be ordered even following a normal chest radiograph when clinical suspicion of an apical lung tumor remains high [15]. It can be further argued that high-resolution CT of the thorax is warranted as initial imaging in such cases, to facilitate timely diagnosis and improve patient outcomes. The recommended investigations for MPM include initial CT of the thorax with contrast, with positron emission tomography-computed tomography (PET-CT) aiding staging and further management [16]. Definitive diagnosis requires biopsy and subsequent histology and immunohistochemical analysis, which remain the gold standard.

## Conclusions

This case contributes to the limited literature documenting the association between MPM and Pancoast syndrome. The development of preganglionic Horner's syndrome in a patient with prior asbestos exposure served as a key clinical indicator that prompted further investigation. Key lessons include the importance of maintaining a high index of suspicion for thoracic malignancy in patients with known asbestos exposure presenting with persistent respiratory symptoms, particularly when accompanied by neurological signs suggestive of apical involvement. Although the primary diagnosis in this case was MPM, a significant smoking history should also be considered a red flag warranting prompt CT in patients presenting with Pancoast syndrome, as it is a major risk factor for apical lung tumors in general. The limitations of chest radiography in detecting apical pathology highlight the need for radiologist review and further cross-sectional imaging when clinical suspicion remains high, even in the context of normal radiographs.

For clinicians managing patients with occupational asbestos exposure, this case emphasizes the importance of recognizing Pancoast syndrome as a potential presentation of apical MPM. Early recognition through initial CT imaging may facilitate more timely diagnosis and potentially improve patient outcomes.
